# Innovative PBAT/PU/MMT@ZnO-NPs bionanocomposite films based on modified montmorillonite for active food packaging

**DOI:** 10.1038/s41598-026-43972-0

**Published:** 2026-04-06

**Authors:** Islam El-Nagar, Ahmed M. Youssef, Tawfik A. Khattab, Hamada S. A. Mandour, El-Refaie Kenawy, A. A. Abd El-Hakim

**Affiliations:** 1 Packaging Materials Department, National Research Centre, 33 El-Bohouth St. (former El-Tahrir St.), Dokki, Giza, 12622 Egypt; 2 Center for Converging Sciences and Emerging Technology (CoSET), Benha National University (BNU), Al Obour, 13518 Egypt; 3 Dyeing, Printing and Auxiliaries Department, National Research Centre, Cairo, 12622 Egypt; 4 Polymer Research Group, Chemistry Department, Faculty of Science, Tanta University, Tanta, 31527 Egypt; 5 Al-Salam University, Cairo Alexandria Agricultural Road, Tanta, Egypt

**Keywords:** Polyurethane, Poly (butylene adipate-co-terephthalate), ZnO-NPs, Montmorillonite, Bionanocomposite, Active packaging, Chemistry, Environmental sciences, Materials science, Nanoscience and technology

## Abstract

Egypt’s plastic garbage problem is becoming worse. The fabrication of biodegradable polymers is an inventive way to decline this pollution. The objective of the current work was to prepare the active packaging bionanocomposites films based on biodegradable poly(butylene adipate-co-terephthalate (PBAT), Polyurethane (PU) and organically modified layered silicates montmorillonite (MMT) containing Zinc oxide nanoparticles (ZnO-NPs) by different concentration (2.5%, 5%, 10%) to improve mechanical and antimicrobial properties of the created bionanocomposite films. Furthermore, the manufactured Poly(butylene adipate-co-terephthalate(,Polyurethane, Montmorillonite, ZnO nanoparticles (PBAT/PU/MMT@ZnO-NPs) bionanocomposite films were assessed using FT-IR, TGA, XRD, SEM, TEM and mechanical properties. Moreover, the barrier properties including water vapor transmission rate (WVTR) in addition the oxygen transmission rate (OTR) of the achieved (PBAT/PU/MMT@ZnO-NPs) bionanocomposite films were evaluated. The fabricated bionanocomposite films demonstrated proper thermal stability. The results showed that adding modified MMT incorporating ZnO-NPs to the PBAT/PU matrix improved the mechanical characteristics, biodegradation, and antibacterial activity. For potential uses in food packaging, the current study provides extremely exciting opportunities for sustainable PBAT/PU-based renewable and antibacterial films.

## Introduction

Recently, scientists and researchers have been focusing on creating new polymeric composites with inorganic nanoparticles to create polymeric nanocomposites for usage in more food applications, like packaging^1,2^. The majority of environmental issues are actually brought on by the use of industrial plastic, which prompts scientists to create sustainable alternatives to address the issue of conventional plastic, particularly in packaging applications. Because they are environmentally friendly, biodegradable, and renewable, biopolymers have drawn a lot of interest as a possible substitute for traditional plastic materials^3^. The majority of fresh products has a limited shelf life and might spoil while being stored and transported. As a result, packaging with efficient technologies must be used, and plastic packaging is a wise choice because it is inexpensive^4^. Semiconductors and metal nanoparticles have special properties. Polymeric materials function as efficient matrix for nanoparticles in the majority of substances^5^. These materials have extraordinary qualities not found in other compounds because of their high surface-to-volume ratio^6^. Due to its unique qualities in comparison to conventional petrochemical-based plastics, green packaging made of biodegradable composite materials has recently attracted a lot of attention from a variety of sectors^7,8^. Additionally, they are completely biodegradable, breaking down into humus, CO_2_, and water. These characteristics can increase the likelihood that they will be used in a variety of applications, including medicine delivery, composting, bio-membranes for wastewater, and intelligent nano-food packaging^9–11^. Therefore, the primary goal of packaging materials is to increase food safety and quality while increasing its shelf life^3,12^.

Biomedical fields, water remediation membranes, automobiles, cables, coatings, furniture cushioning foams, and food packaging are just a few of the many industrial uses for thermoplastic polyurethane (TPU) elastomer, a biodegradable polymer that has drawn significant attention from a variety of disciplines due to its unique chemical and physical characteristics^13–15^. Because of its mixed plastic and elastomer properties that are comparable to those of other conventional plastics, TPU has both soft (linear long chains of diols) and hard (diisocyanate and short-chain molecules) segments in its backbone^16,17^.

As a result of the increasing need for sustainable and natural food packaging products, biodegradable polymers are the subject of extensive research^18,19^. But one particularly interesting substance is poly (butylene adipate-co-terephthalate) (PBAT), a biodegradable aliphatic-aromatic copolyester known for its resilience, pliability, and compostability^20^. PBAT is currently a desirable option for use in food packaging and a desirable substitute for traditional petroleum-based plastics because it decomposes naturally. Despite PBAT’s superior mechanical and biodegradable qualities, it lacks the barrier and thermal stability needed for effective food packaging^21^.

Because of its high gas transmission, low heat distortion temperature, high elongation at break, minimal melt viscosity, and substantial flexibility, a biodegradable polymer like poly (butylenes adipate-co-terephthalate) (PBAT) is useful for food biodegradable packaging materials^22–24^. One well-known and often utilized clay is montmorillonite (MMT). It is a 2D layered silicate that is electrically neutral and made up of many cations as well as a distinct layer of negative charges. Materials can be intercalated into MMT’s layers thanks to its remarkable adsorption capabilities in addition to its water-swelling capabilities^25^.

The exceptional combination properties of polymer/clay nanocomposites (PCNs) have garnered significant interest from academics as well as industry. These properties include enhanced mechanical characteristics, a significant rise in thermal stability, fire retardancy for flammability, and a decline in gas permeability^26–30^. At the moment, exposing the polymer matrix to UV light prior to burial has shown a considerable improvement in PBAT biodegradability when mixed with clay^31^.

Researchers investigated at developing nanocomposites by incorporating nanoparticles into PBAT in an effort to get over the limitations^32,33^. These PBAT-based nanocomposites’ mechanical strength, thermal stability, and gas and moisture barrier qualities are improved by the addition of nanoparticles such as metal or metal oxide nanoparticles, graphene oxide, cellulose nanocrystals, and nanoclays^34,35^. By decreasing their permeability to oxygen and water vapour, nanoparticles added to PBAT matrices improve the performance of the packaging material and extend the shelf life of food goods^36^. Additionally, specific nanoparticles contain antimicrobial properties that improve the safety and preservation of food. The creation of PBAT nanocomposites reflects the increased attention being paid to reducing plastic waste globally while preserving the qualities and characteristics needed for use in food packaging^37^.

Since the layered clay is an element that occurs naturally that breaks down without damaging the soil, biodegradable matrix is regarded as sustainable^22^. The issues with neat PBAT may be resolved by the PBAT/organoclay complex, which could also improve several of its characteristics for usage in a variety of applications. These improvements rely on the kind in addition structure of the organoclay utilized accompanied by how the organoclays are distributed throughout the polymer matrix^38–40^. Because of its unique characteristics, which include antibacterial properties, chemical stability, catalytic activity, reduced toxicity^28,41^, wide band gap, high refractive index, and ultraviolet absorption, scientists have been using zinc oxide nanoparticles (ZnO-NPs) extensively in recent years^26,27,42,43^. By preparing nanohybrides, these properties can be ascertained. Furthermore, ZnO-NPs aggregate easily in the mixed configuration process due to their high surface energy. Moreover, to achieve the desired qualities of hybrid materials, a good, regular dispersion of inorganic filler with host materials is highly suited^29,44^.

In the current manuscript we prepared polymer bionanocomposite based on nanomaterials (MMT and ZnO-NPs) and biopolymer (PU and PBAT). The goal of this contribution is to investigate the effect of (MMT and Zno-NPs) on the physical, gas barrier, thermal, mechanical, antimicrobial properties and morphology of PU/PBAT/organoclay/ZnO bionanocomposites.

## Materials and methods

### Materials

Pellets of polybutylene adipate terephthalate (PBAT) with a melting point of 110–120 °C, density was (1.25 g/cm^2^) were acquired from BASF, Germany. The thermoplastic polyurethane (TPU)) was supplied by BASF Germany with material details (Physical form (Pellets), Density (1.2 g/cm^3^) at 21 °C, Melt Index around 3–6 g/10 min using (210 °C, 1.2Kg) and the shore hardness about 75 A). Southern Clay Products delivered the montmorillonite nanoclay, whereas Siga-Aldrich Chemicals (Cairo, Egypt) provided Cloisitew 30B (MMT-OH), zinc-acetate (Zn (CH_3_COO)_2_·2H_2_O), Tween 80, glycerol, and Tetrahydrofuran (THF) and Acros provided the sodium hydroxide (NaOH) and pure ethanol. All chemicals were of analytical grade.

### Preparation of ZnO nanoparticles

Zinc acetate and a base (NaOH) reacted to create ZnO nanoparticles in an alcoholic media. In this study, 1 L of absolute ethanol was used to dissolve 3.96 g of zinc acetate and 1.5 g of NaOH, which were then refluxed for one hour at 70 °C. Zinc acetate was changed into zinc oxide via the reaction of the acetate group with NaOH. The produced ZnO-NPs, which were distributed in an alcohol medium, were clear and transparent and could remain stable for at least two weeks. Following the process, DI-water was added to the zinc oxide that had been dissolved in ethanol in order to purify it. The ZnO nanoparticles were then repeatedly centrifuged at 8000 rpm for 8 min in order to remove them from the dispersion supernatant. In order to obtain ZnO-NPs water dispersion, the ZnO nanoparticles were finally dispersed in DI-water.

### Manufacturing of the bionanocomposite packaging films

The manufacture of the active packaging films (PBAT/PU/MMT@ZnO-NPs) was concisely produced by adding different concentration (2.5%, 5%, and 10%) of ZnO-NPs. To prepare PBAT/PU/MMT, PBAT and TPU were dissolved in Tetrahydrofuran (THF) at a concentration of 5% (wt/v), MMT was added at a rate of 2% (wt/wt) based on the solid content of PBAT/PU, and the mixture was sonicated for 15 min using an ultrasonic probe. ZnO-NPs were added to PBAT/PU/MMT separately at room temperature in concentrations of 2.5%, 5%, and 10% to enhance the mechanical properties of bioactive films. The mixture was stirred for three hours at 1500 rpm. The prepared PBAT/PU/MMT mixture with different concentration of ZnO-NPs in addition to PBAT/PU were cast inside perti-dishes then air dried (Table 1).

**Table 1:** The recipe of the fabrication of PBAT/PU/MMT@ZnO-NPs bionanocomposite films.

Samples	Cods	PU, wt%	PBAT, wt%	MMT, wt%	ZnO-NPs, wt%
PU	B1	10	0	0	0.0
PBAT/PU	B2	5	5	0	0
PBAT/PU/MMT	T0	5	5	2	0.0
PBAT/PU/MMT	T1	5	5	2	2.5
PBAT/PU/MMT	T2	5	5	2	5
PBAT/PU/MMT	T3	5	5	2	10

### Evaluation of fabricated active films

Several tools were used to analyze PBAT/PU, MMT, and the produced bionanocomposite films.

#### X-ray diffraction (XRD)

The X-ray diffractometer was used to estimate the XRD patterns of the bioplastic films. The Cu Kα radiation source (45 kV, 40 mA, = 0.15418 nm) was used with a Philips X-ray diffractometer (PW1930 generator, PW 1820 goniometer) in a 2θ range of 5°–80°. The time was set to one second, and the step size was set to 0.02^22,26^.

#### Transmission electron microscopy (TEM)

The morphology of the manufactured bionanocomposite was studied via transmission electron microscope (TEM), JEM-1230; JEOL Ltd., Tokyo, Japan, at an accelerating voltage of 80 kV^22,26^.

#### FT-IR spectroscopy

The produced bionanocomposite with varying ZnO-NP ratios (2.5%, 5%, and 10%) had FT-IR spectra collected in the 500–4000 cm^− 1^ range using a Shimadzu 8400 S^26^.

#### Mechanical properties of the prepared bionanocomposite films

The mechanical properties of the produced bionanocomposite films have been assessed utilizing INSTRON 34SC-5 testing machine accordance with ASTM Standard D638-91. The testing apparatus was operated at a speed of 10 mm/min with a 5 K N load cell.

#### Barrier properties

A GBPI W303 (B) Water Vapour Permeability Analyser was used to test the water vapour transmission rate using the cup technique. The amount of water vapour that has passed through a unit area in a unit of time under particular humidity (4–10%) and temperature 38 °C is the water vapour transmission rate (WVTR), as determined by the 53,122–1 standards, JIS Z0208, TAPPI T464, ISO 2528, ASTM D1653, and ASTM E96. Additionally, the rate of gas gearbox GTR (O_2_) is measured using the N530 B Gas Permeability Analyzer (China) in accordance with ASTM D1434-82 (2003) specifications.

#### TGA test

The developed bionanocomposites samples were subjected to thermal properties examination, which included Thermogravimetric analyses (TGA). The SDT Q600 thermal analyzer, manufactured in the USA, was used to do the analysis in a nitrogen atmosphere at a heating rate of 10 °C/min.

### Microorganism’s strains

Microorganism’s strains were obtained from the Dairy department at National Research Centre, Egypt as: *Escherichia coli* ATCC 8739, *Bacillus cereus* ATCC 33018, *Listeria monocytogenes* ATCC 5980, *Staphylococcus aureus* ATCC 6538, *Salmonella Typhimirum* ATCC 14028, and *Aspergillus niger* ATCC 10404.

### Antimicrobial activity of the bionanocomposites films loading ZnO-NPs concentrations

According to BSAC (2007), the antimicrobial activity was assessed by selecting an identical colony from either mold extract agar (fungi strains) or nutrient agar medium (bacteria strains) following inoculation with individual strains and overnight incubation. Five milliliters of tryptone soy broth were then added to the selected colonies, and they were incubated at 35 °C for bacteria and 25 °C for fungi until the visible turbidity reached 0.5 “McFarland” standard solution. Mueller-Hinton agar plates were then used to test the antimicrobial properties of the bionanocomposites films. 25 milliliters of the agar medium were added to the sterilized plates, and the plates were allowed to harden. Using a sterile cotton swab, a particular strain was applied to each plate, covering the surface of the agar medium. The infected plates were then let to rest for 15 min. The various films of bionanocomposites containing ZnO-NPs at concentrations of 0.0, 2.5, 5.0, and 10% were cut into disks of 6 mm in diameter and carefully positioned on the inoculation plates. Following an overnight incubation period at 35 °C for bacteria and 25 °C for fungus, the inhibition zone diameters (mm) surrounding each disk were measured as millimeters.

### Statistical analysis

The manufactured bionanocomposites films were examined using was performed using the Statistical Analysis System using the ANOVA procedure for analysis of variance, and the general linear model (GLM) procedure for SAS software (SAS, 1990). The results were expressed as mean ± standard deviation and the differences between means were tested for significance using Duncan’s multiple range tests at (*p* ≤ 0.05). Three replicates’ worth of data is shown as mean ± SD.

## Result and discussions

### X-ray diffraction (XRD)

**Fig. 1:**
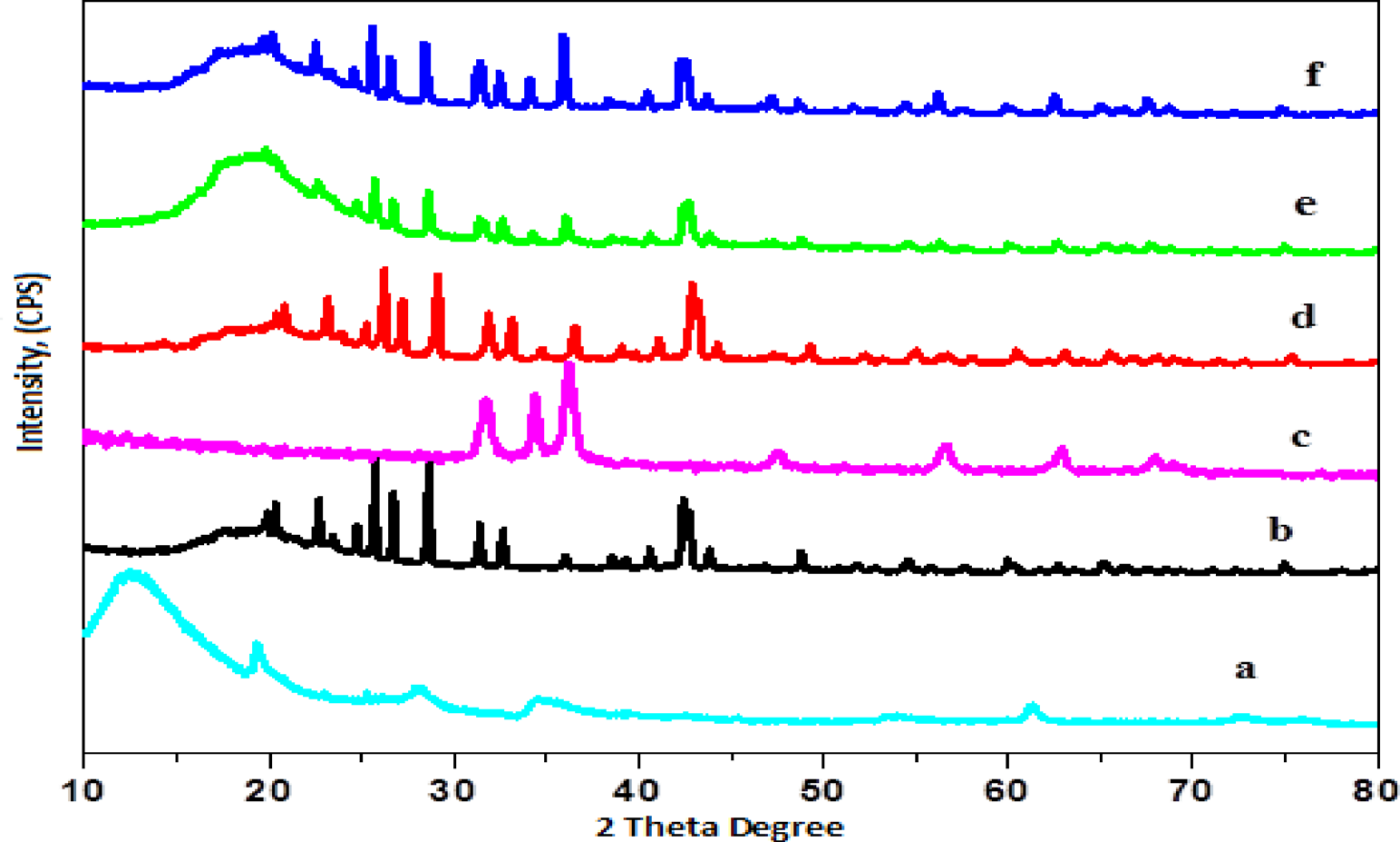
XRD of (**a**) MMT, (**b**) PBAT/PU/MMT, (**c**) ZnO-NPs, as well as PBAT/PU/MMT-ZnO Bionanocomposite with different concentration of ZnO-NPs, (**d**) 2.5, (**e**) 5, and (**f**) 10%.

The XRD of MMT, PBAT/PU/MMT, ZnO-NPs, as well as PBAT/PU/MMT-ZnO bionanocomposite with different concentration of ZnO-NPs were reveals in (Fig. 1) the XRD patterns for the neat MMT displayed characteristic peaks at 2θ = 12.4°, 19.6°, 28.8°, 34.6°, 62.2°, and 74.5° as shown in (Fig. 1a). Additionally, the X-ray diffraction (XRD) patterns of ZnO-NPs nanoparticles match those on JCPDS Card No. 36-1451. With lattice constants of a = 3.249 Å and c = 5.206 Å, it validates the crystalline nature and hexagonal phase (wurtzite structure) of ZnO-NPs. The (100), (002), (101), (102), (110), (103), (112), (201), and (202) planes of ZnO-NPs can be linked to the reflections seen at 31.6°, 34.8°, 36.5°, 48.2°, 56.4°, 62.6°, 68.1°, 69.3°, and 76.8°. The strong peak at 2θ = 11.2° (7.96 Å) nearly vanished after loading varying concentrations of ZnO-NPs (2.5, 5.0, and 10.0%) with the PBAT/PU/MMT nanocomposites matrix. The other diffraction peaks in the matrix were comparable to those of pure MMT and the fabricated ZnO-NPs in the PBAT/PU matrix, and the results stayed the same as shown in (Fig. 1d, e, and f).

The significant decrease or disappearance of the basal reflection in our system points to a highly disordered or exfoliated intercalated structure rather than straightforward ZnO surface adsorption. ZnO nanoparticles in MMT galleries probably cause interlayer spacing to increase and interlayer van der Waals interactions to weaken, which promotes delamination in the PBAT/PU matrix. Subsequently ZnO ZnO-NPs incorporation, the 2θ ≈ 11.2° peak disappears, indicating that ZnO-NPs were effectively incorporated into or separated from MMT layers. • ZnO interferes with MMT’s normal lamellar stacking. MMT clay delamination is improved by subsequent blending into PBAT/PU because of polymer chain penetration. This observation therefore favors strong interfacial interaction and enhanced nanoscale dispersion over straightforward physical mixing.

Additionally, ZnO incorporation affects matrix crystallinity by promoting structural disorder, reducing long-range ordering, and acting as a nucleating agent, alongside changes in hydrogen bonding and polymer chain mobility. Overall, the results demonstrate successful ZnO loading in MMT layers, structural disruption, and altered crystallization behavior within the PBAT/PU matrix.

### Transmission electron microscopy (TEM)

**Fig. 2:**
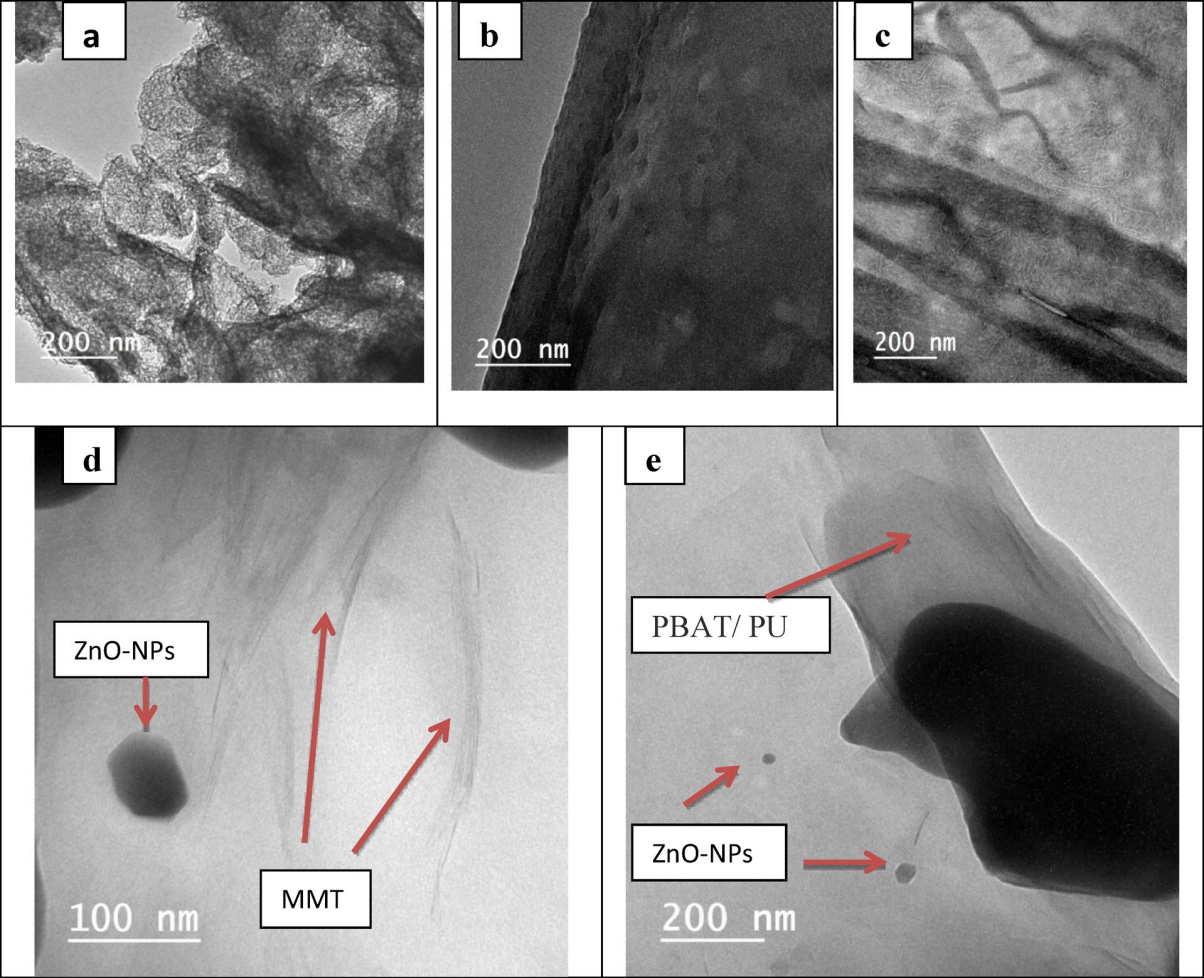
TEM image of (**a**) MMT, (**b**) B2, (**c**) T0, (**d**) T1 and (**e**) T3.

The surface morphology of PBAT/PU blend and its bionanocomposite films was characterized by TEM. Figure 2 displays the pure MMT, PBAT/PU/MMT and the prepared PBAT/PU/MMT-ZnO Bionanocomposite. The dark layered represents the MMT nanoparticle distributed in the PBAT/PU matrix. These particles are very similar in shape to the original clay tactoid in the PBAT/PU nanocomposites. The interaction between the ZnO nanoparticles and the PBAT/PU/MMT bionanocomposite matrix is well revealed from the TEM images shown in (Fig. 2d and e). The ZnO-NPs in the PBAT/PU matrix are of spherical shape randomly distributed for 2.5 wt% of ZnO-NPs in bionanocomposite films.

### Fourier transforms infrared (FTIR) spectroscopy

**Fig. 3:**
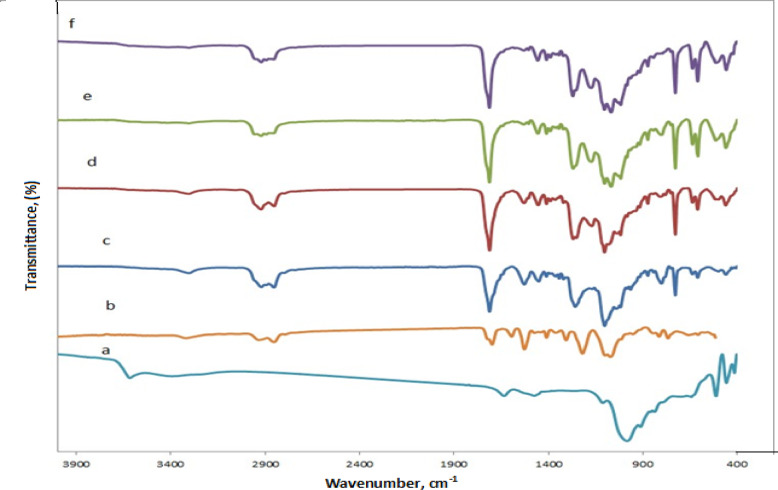
FTIR of (**a**) MMT, (**b**) Pure PU, (**c**) PABT/PU/MMT, as well as PBAT/PU/MMT-ZnO-NPs Bionanocomposite with different concentration of ZnO-NPs, (**d**) 2.5, (**e**) 5, and (**f**) 10%.

The FTIR spectra of the MMT, Pure PU, and the fabricated PABT/PU/MMT, as well as PBAT/PU/MMT-ZnO-NPs bionanocomposite containing different loadings of ZnO-NPs, (2.5, 5, and 10%) were depicted in (Fig. 3). As shown in (Fig. 3a), the MMT exhibits OH around 3241 cm^− 1^ and Si–O–Si groups approximately 1060 cm^− 1^, which may serve as hydrogen-bonding sites for the functional groups of the PABT/PU matrix. The polyurethane’s (PU) FTIR spectrum is seen in (Fig. 3b). The urethane carbonyl band was identified at 1708 cm^− 1^, whereas the –CH_2_– stretching bands are seen at 2925 and 2856 cm^− 1^. The presence of the interaction between the hydroxyl group and the isocyanate is indicated by the C–N stretching bands, which are usually observed at 1520 and 1445 cm^− 1^ together with those of the N–H bending in the plane. At 1079 cm^− 1^, the C–O–C characteristic band was found. The PBAT’s C–H stretching vibration is represented by the absorption peak, which is located at 2954 cm^− 1^. The C=O stretching vibration is indicated by the peak at 1715 cm^− 1^. The stretching vibration of C–O–C is indicated by the peaks at 1268 and 1100 cm^− 1^. Additionally, as seen in (Fig. 3c), also, a large absorption peak at 723 cm^− 1^ absorbs closer to –CH_2_ groups.

Additionally, FTIR spectra of ZnO nanoparticles usually exhibit distinctive peaks associated with Zn–O stretching vibrations and other functional groups, depending on the synthesis technique and surface modifications, when ZnO-NPs are added with varying loadings. The Zn–O bond (about 400–600 cm^–1^) and O–H stretching (approximately 3350 cm^–1^) caused by adsorbed water or surface hydroxyl groups are common peaks. The existence of organic molecules adsorbed on the surface of the nanoparticle may be indicated by additional peaks. Correspondingly, the Fig. 3d, e and f are the same due to the presence of MMT and ZnO-NPs onto the PABT/PU matrix.

### Evaluation of the mechanical properties of the prepared bionanocomposite films

Table 2 displays the mechanical properties of the prepared bionanocomposite films, particularly PBAT/PU/MMT loaded with varying concentrations of ZnO-NPs, revealing significant insights into the material performance as a function of nanoparticle loading. The data shows that the elongation at the break percentage varies significantly (*p* < 0.05) with different ZnO-NP concentrations. The film with 2.5% ZnO-NPs demonstrates the greatest elongation at 365.13%, indicating that this concentration optimally enhances the flexibility of the composite. In contrast, the samples with 0.0% and 5% ZnO-NPs show lower elongation values (257.5% and 228.7%, respectively), indicating a decrease in ductility. Notably, the film with the highest ZnO-NP concentration (10%) shows a marked reduction in elongation (144.3%), suggesting that excessive ZnO-NP content may lead to a more brittle structure. This trend emphasizes the importance of optimizing nanoparticle concentration to balance flexibility and strength in composite materials (31%), this may be attributed to the agglomeration of nanoparticles, leading to increased brittleness.

**Table 2:** The mechanical properties of the prepared bionanocomposites films loading ZnO-NPs via different concentration.

Samples	ZnO-NPs, %	Elongation, %	Tensile strength (MPa)	Young’s modulus (MPa)
PBAT/PU/MMT	0.0	257.5 ± 0.05^b^	7.35 ± 0.01^a^	56.95 ± 0.04^d^
PBAT/PU/MMT	2.5	365.13 ± 0.03^a^	7.02 ± 0.10^b^	68.3 ± 0.05^c^
PBAT/PU/MMT	5	228.7 ± 0.05^c^	6.39 ± 0.05^c^	72.5 ± 0.07^b^
PBAT/PU/MMT	10	144.31 ± 0.01^d^	5.11 ± 0.04^d^	75.38 ± 0.03^a^

Data expressed as average of three replicates. Means in the same column showing the same small letters are not significantly different (*p* > 0.05).

Tensile strength also demonstrates variation with ZnO-NP loading. The tensile strength decreases (*p* < 0.05) from 7.35 MPa at 0% ZnO-NPs to 5.11 MPa at 10% ZnO-NPs. This trend suggests that while lower concentrations of ZnO-NPs do not significantly compromise the tensile strength, higher concentrations may negatively impact the load-bearing capacity due to the aforementioned agglomeration effect, which disrupts the uniform stress distribution within the matrix. Young’s modulus, representing the stiffness of the material, increases with the addition of ZnO-NPs. The modulus rises from 56.95 MPa at 0% ZnO-NPs to 75.38 MPa at 10%. This increase indicates that the bionanocomposite becomes stiffer with higher ZnO-NP content, which may enhance its structural integrity. However, the trade-off between stiffness and flexibility must be carefully considered, as the increased stiffness could lead to a decrease in elongation and overall ductility. All in all, the concentration of ZnO-NPs plays a crucial role in balancing flexibility and strength. The optimal concentration appears to be around 2.5%, where elongation is maximized, while higher concentrations lead to increased stiffness at the expense of tensile strength and ductility.

The improvement in elongation at break at 2.5 wt% ZnO-NPs loading is attributed to enhanced ZnO/MMT and PBAT/PU matrix interaction, better stress transfer from uniform nanoparticle dispersion, increased interfacial adhesion through hydrogen bonding, and slight plasticization effects. Low loading allows well-dispersed nanoparticles to improve stress transfer, while excessive loading leads to agglomeration, stress concentration points, less effective interfacial bonding, and restricted polymer chain movement. This is evidenced by the decrease in elongation and tensile strength at higher ZnO-NPs concentrations. Also, effective mechanical reinforcement is influenced by dispersion quality, surface adhesion, and optimal filler loading, with optimal performance observed below the aggregation threshold of 2.5 wt%. the obtained data indicates that 2.5 wt% is the optimal level for dispersion and interfacial interaction, while higher loadings lead to nanoparticle agglomeration, stress concentration, and decreased mechanical performance. The mechanical behavior is a result of the balance between reinforcement and aggregation effects.

### Evaluation of the Barrier properties of the prepared bionanocomposite films

Table 3 indicates that the incorporation of ZnO-NPs into PBAT/PU/MMT films significantly (*p* < 0.05) influences both OTR and WVTR. The OTR values show a clear upward trend with increasing concentrations of ZnO-NPs. The film containing 0.0% ZnO-NPs exhibits an OTR of 2.85 cc/(m^2^.day), which increases to 3.66 cc/(m^2^.day) at 2.5% ZnO-NPs. However, a dramatic increase is observed at higher concentrations, with OTR values soaring to 157.83 cc/(m^2^.day) and 217.21 cc/(m^2^.day) for 5% and 10% ZnO-NPs, respectively. This substantial increase suggests that higher nanoparticle concentrations may create pathways or defects within the polymer matrix, enhancing gas permeability. This could be advantageous in specific applications where increased gas exchange is desired but may compromise the barrier properties for certain packaging applications. In contrast to the OTR, the WVTR values exhibit a decreasing trend (*p* < 0.05) as the concentration of ZnO-NPs increases.

The WVTR starts at 137.307 g/(m^2^ day) for the 0.0% ZnO-NP film and decreases to 125.163 g/(m^2^ day) at 2.5%. This reduction continues, with WVTR values declining to 102.511 g/(m^2^ day) and 95.285 g/(m^2^ day) for 5% and 10% ZnO-NPs, respectively. The decrease in WVTR indicates that the addition of ZnO-NPs improves the water vapor barrier properties of the films. This enhancement can be attributed to the potential formation of a more compact and consistent structure within the composite, which restricts water vapor diffusion. Therefore, the results show that while increasing ZnO-NP concentrations improve water vapor resistance, they significantly (*p* < 0.05) enhance gas permeability.

**Table 3:** The gas transmission rate (OTR) and the water vapor permeability (WVTR) of the prepared bionanocomposites films containing (ZnO-NPs) by different concentration.

Samples	ZnO-NPs, %	OTR, cc/(m^2^.day)	WVTR, g/(m^2^ day)
PBAT/PU/MMT	0.0	2.85 ± 0.05^c^	137.307 ± 0.01^a^
PBAT/PU/MMT	2.5	3.66 ± 0.37^c^	125.163 ± 0.05^b^
PBAT/PU/MMT	5	157.83 ± 0.03^c,b^	102.511 ± 0.04^c^
PBAT/PU/MMT	10	217.21 ± 0.05^a^	95.285 ± 0.01^d^

Data expressed as average of three replicates. Means in the same column showing the same small letters are not significantly different (*p* > 0.05).

### Thermal analysis of the prepared bionanocomposite films

**Fig. 4:**
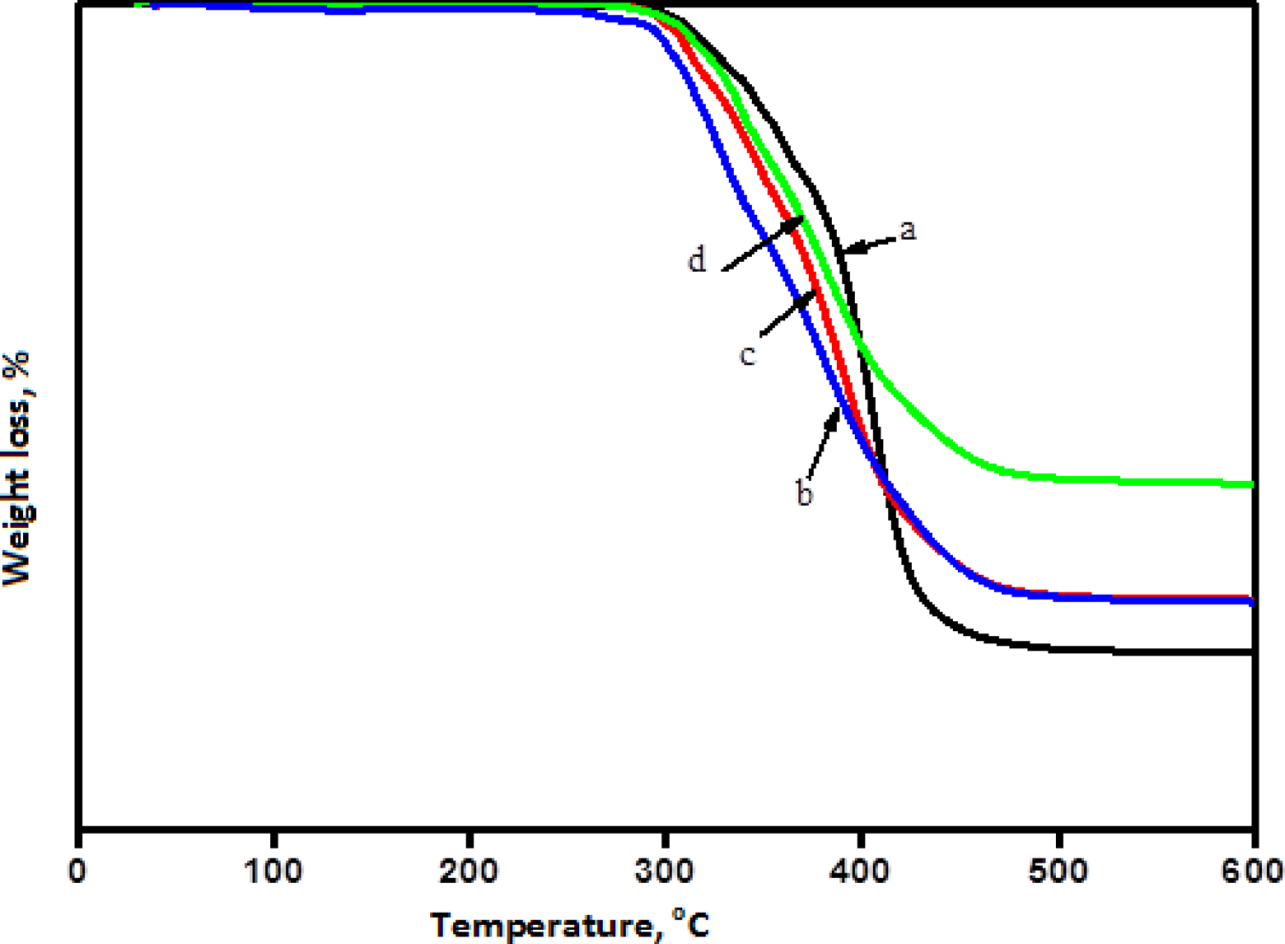
TGA thermograms of (**a**) PBAT/PU/MMT, as well PBAT/PU/MMT-ZnO-NPs bionanocomposite with different concentration of ZnO-NPs, (**b**) 2.5, (**c**) 5, and (**d**) 10%.

Thermogravimetric analysis was used to examine the manufactured PBAT/PU/MMT-ZnO-NPs bionanocomposite’s thermal stability (TGA). When heated to 600 degrees Celsius, the TGA verified that the pristine MMT was extremely stable and did not exhibit any discernible weight loss. The TG mass loss curves for PBAT/PU/MMT and a series of PBAT/PU/MMT-ZnO-NPs bionanocomposite with varying ZnO-NPs concentrations are shown in Fig. 4. The graph for the PBAT/PU/MMT sample indicates an onset temperature of 300 °C and a single degradation phase, which is the breakdown of the polymer backbone. The loss of absorbed water, which started at around 50 °C, was probably the cause of the 2.5 weight% weight loss up to about 100 °C. All PBAT/PU/MMT-ZnO-NPs bionanocomposite samples show a single degradation phase of weight loss between 300 and 450 °C during their thermal breakdown. In comparison to the PBAT/PU/MMT bionanocomposite, all samples show an increase in the beginning temperature of deterioration, indicating improved thermal stability. The following explains how PBAT/PU/MMT-ZnO-NPs bionanocomposites improved in thermal stability. The first is the creation of organo-MMT, which serves as an insulator and mass transport barrier between the polymer and the superficial zone where polymer breakdown occurs. It was discovered that the polymer decomposition temperature (PDT) was 300 °C. The Figure show all experimentally obtained degradation temperatures of the bionanocomposites under study as a function of MMT loading and ZnO-NPs.

### Antimicrobial properties of the fabricated bionanocomposite films

The inhibitory activity of various ZnO-NPs concentrations incorporated into bionanocomposites films against various strains was displayed in (Table 4). When the ZnO-NP-free blank film was first analyzed, it showed no inhibitory antimicrobial activities against the microorganisms that were tested. Inhibition activity against tested strains varied from 6 to 14 mm, depending on the type of strain, at a concentration of 2.5% ZnO-NPs inside coated film. The ZnO-NPs concentration of 5.0%, however, demonstrated superior antimicrobial activity against the strains that were tested. Depending on the strain type, the inhibition zones’ diameters varied from 11 to 18 mm. Additionally, the other high concentration of 10% ZnO-NPs more boosted the diameter of the inhibitory zone, which was measured to be between 15 and 23 mm. Additionally, the data showed that for all films with ZnO-NPs concentrations, the strains that were more susceptible were *S. Typhamirum*, *E. coli*, and *B. cereus*.

**Table 4:** Antimicrobial activity of the bionanocomposite films loading ZnO-NPs concentrations.

Tested strains	Bionanocomposites films with different ZnO-NPs concentrations (%)			
	Blank	2.5	5.0	10.0
Diameter of inhibition zone (mm)				
*S. typhamirum*	N.D	10 ± 0.7^Cc^	13 ± 0.5^Cb^	22 ± 1.0^Aa^
*E. coli*	N.D	12 ± 0.5^Bc^	16 ± 1.0^Bb^	23 ± 0.5^Aa^
*S. aureus*	N.D	11 ± 1.2^Cc^	14 ± 0.2^Cb^	19 ± 0.2^Ba^
*B. cereus*	N.D	14 ± 0.7^Ac^	18 ± 1.0^Ab^	23 ± 0.5^Aa^
*L. monocytogenes*	N.D	08 ± 0.4^Dc^	12 ± 0.2^Db^	17 ± 0.7^Ca^
*A. niger*	N.D	07 ± 0.2^Dc^	11 ± 0.5^Db^	15 ± 1.0^Da^

Data expressed as mean of three replicates. Means between columns showing the same capital letters are not significantly different (*p* ≤ 0.05). Means between rows showing the same small letters are not significantly different (*p* ≤ 0.05).Data expressed as (mean ± SD) of 3 replicates.

Generally, nanocomposites’ had ability to increase surface area and special qualities have made these materials with promise for antimicrobial applications. Silver, copper, or zinc oxide nanoparticles are incorporated into a polymer matrix to create nanocomposites, which have exceptional antimicrobial action against a variety of pathogen strains^45,46^. The physical and chemical interactions between the composite material and microbial cells are the main ways that ZnO-NPs nanoparticles exhibit antimicrobial properties. The inherent characteristics of ZnO nanoparticles can cause microbial cell membranes to rupture, resulting in cell leakage and eventual cell death as mentioned by previous studies^47,48^. Zinc oxide (ZnO) serves two purposes in food packaging: it improves texture, appearance, and storage qualities while also acting as a photocatalytic agent to break down organic compounds and bacteria. ZnO’s antimicrobial properties, improved material barrier, and hydrophobicity are the reasons for its effectiveness in food packaging^49^.Generally, food packaging materials are supplemented with nanomaterials such as metal/metal oxide nanoparticles (NPs), carbon nanotubes, and nanoclays to provide their antibacterial, antifungal, ethylene scavenging, and oxygen/water vapor absorption properties^50^.According to^51^ found that acyclovir, nano-chitosan, clove oil with ZnO-NPs nanocomposite-based cotton textiles shown promising antibacterial action against *S. aureus*, *S. pyogenes*, *E. coli*, and *K. aerogenes*.

## Conclusions

In this work, PBAT, PU matrix, and MMT/ZnO nanoparticles are utilized as reinforcing agents to create durable films utilizing the solution casting technique. The current work aimed to produce active packaging bionanocomposite films based on biodegradable poly (butylene adipate-co-terephthalate, or PBAT), polyurethane (PU), and organically modified layered silicates montmorillonite (MMT) containing zinc oxide nanoparticles (ZnO-NPs) at varying concentrations (2.5%, 5%, and 10%) in order to improve the mechanical and antimicrobial capabilities of the PBAT/PU/MMT-ZnO-NPs bionanocomposite films. The generated poly(butylene adipate-co-terephthalate), polyurethane/montmorillonite, and ZnO nanoparticles (PBAT/PU/MMT@ZnO-NPs) bionanocomposite films were further assessed using FT-IR, TGA, XRD, SEM, TEM, and mechanical properties. The antibacterial activity of the films against *S. aureus*, *S. pyogenes*, *E. coli*, and *K. aerogenes* increased with the addition of MMT/ZnO nanoparticles. Out of all the films with varying weight percentages, the PBAT/PU/MMT-ZnO-NPs bionanocomposite film has been shown to be the most effective for food packaging. Compared to PBAT/PU/MMT, the PBAT/PU/MMT-ZnO-NPs bionanocomposite films exhibited the largest decreases in WVTR as ZnO-NPs concentration increased, while the OTR increased as ZnO-NPs concentration increased. This indicates that PBAT/PU/MMT-ZnO-NPs films are more valuable and useful for a variety of applications.

## Data Availability

The datasets generated during and/or analyzed during the current study are available from the corresponding author on reasonable request.
